# Factors influencing the patient experience of gastrointestinal endoscopic ultrasound: a Swedish cross-sectional study

**DOI:** 10.1007/s00464-026-12777-7

**Published:** 2026-05-15

**Authors:** Jessica Ryhlander, Gisela Ringström, Björn Lindkvist, Per Hedenström

**Affiliations:** 1 Department of Molecular and Clinical Medicine, Institution of Medicine, Sahlgrenska Academy, University of Gothenburg, Gothenburg, Sweden; 2 Division of Medical Gastroenterology, Department of Internal Medicine, Sahlgrenska University Hospital, Blå Stråket 3, 41345 Gothenburg, Region Västra Götaland Sweden

**Keywords:** Endosonography, Discomfort, Pain, Patient-centered care, Patient care management

## Abstract

**Introduction:**

Although the technical and diagnostic quality of gastrointestinal endoscopic ultrasound (EUS) has been thoroughly studied, research on the patient experiences of EUS remains limited. This study aimed to identify factors influencing the patient’s experience of EUS performed during conscious sedation.

**Materials and methods:**

Between September 2020 and December 2021, a cross sectional study was conducted at a high-volume, tertiary endoscopy centre in western Sweden. All outpatients aged > 18 years who underwent EUS were eligible for inclusion. Participants completed a study-specific questionnaire for Patient-Reported Experience Measures (PREM), assessing pre-procedure anxiety, procedure pain, and discomfort of EUS, using the Visual Analogue Scale (VAS). Patient- and procedure related factors were analysed in relation to VAS scores as predictors of patient experience.

**Results:**

A total of 306 patients (median age 69 years; 163 women) were included. PREM indicated care as dignified and respectful. Median VAS scores were pre procedure anxiety, 24 (IQR 7–55); procedural pain, 7 (IQR 3–18); and procedural discomfort, 10 (IQR 3–22). A previous negative endoscopy experience was the strongest predictor of higher preprocedural anxiety, followed by female gender. These factors were also common among patients reporting higher levels of procedural pain and discomfort.

**Conclusion:**

In general, routine EUS is well tolerated by patients with minimal pain and discomfort during conscious sedation. However, previous negative experiences and female gender were associated with higher pre-procedure anxiety and greater procedural discomfort. These findings stress the importance of personalized care, where patients at increased risk should be offered tailored information and supportive strategies.

**Graphical abstract:**

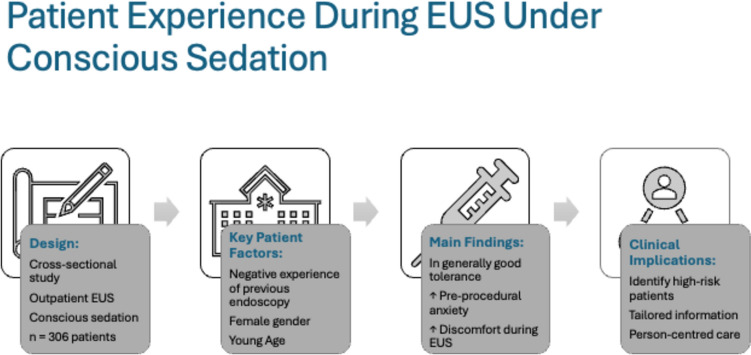

**Supplementary Information:**

The online version contains supplementary material available at 10.1007/s00464-026-12777-7.

The patient experience is a crucial dimension of healthcare, alongside patient safety and clinical effectiveness [1]. Gastrointestinal (GI) endoscopic ultrasound (EUS) has become a widely used technique to investigate pathology in the GI tract [2]. The EUS procedure is well-established for routine screening and monitoring of malignancies and therapeutic purposes [3]. EUS is technically complex, may be time-consuming, and the use of a large, rigid-tipped endoscope can cause discomfort or pain for patients [4]. In certain individuals, endoscopy can evoke emotions such as fear, stress, anxiety, embarrassment, and inferiority [5–7]. A detailed understanding of patient characteristics associated with poor procedure tolerance is important in enabling the endoscopy team to take preventive measures [8]. Patient experience contains more than just tolerance to the procedure, such as easy access to information, effective communication, and dignified, safe, and respectful care [9, 10]. Furthermore, to meet international standards for high-quality GI endoscopy and patient needs, healthcare regularly needs to be evaluated and revised.

Current knowledge of patient experience of EUS is largely based on studies on mixed populations of patients undergoing EUS or endoscopic retrograde cholangiopancreatography (ERCP), in in- or outpatient care and with different types of sedation [11–13]. A large proportion of EUS are performed in the context of surveillance programs of premalignant lesions, and many of these patients are subjected to multiple EUS over time [14]. Previous research has shown that patients undergoing clinical endoscopy monitoring programs find these examinations intrusive, physically burdensome, and some dread the next procedure [6, 7, 15]. Additionally, a poor procedure experience has been shown to evoke aversion in the face of future examinations, which in turn may affect the patient´s compliance with the surveillance protocol and compromise the effectiveness of the program [7, 15]. Furthermore, unbearable pain and discomfort during a previous endoscopy negatively affect patients’ willingness to undergo a repeat examination [6, 7, 16]. Assessing patients’ experiences is crucial to ensure the safety, acceptability and adherence to endoscopic surveillance programs. Therefore, to evaluate the service quality of EUS and identify areas for improvement, it is crucial to measure the patient experience of EUS by adequate means. The aim of this study was to investigate the patient experience of EUS, identify patient- or procedure-related factors that are associated with patient experience, and explore ideas for future improvements in line with person-centred care.

## Materials and methods

### Study design

This was a cross-sectional study with a quantitative design conducted at Sahlgrenska University Hospital, Gothenburg, Sweden, a high-volume referral centre for endoscopy from September 2020 to December 2021. The study was reported in accordance with the STROBE statement (Strengthening the Reporting of Observational Studies in Epidemiology) checklists of items for cross-sectional studies. All study subjects were included consecutively. Written informed consent was obtained, and the patients were examined and managed in accordance with clinical routines. All patients > 18 years referred for EUS as an outpatient procedure were eligible for study inclusion. Patients were excluded if they were examined under general anaesthesia, if they had a cognitive impairment, or if they could not understand or speak the Swedish language.

Data was stored in anonymised form in the study database, with only the main investigator having access to the identification code. The ethical review board in Gothenburg approved the study (registration number 573-09). The study was conducted in line with the World Medical Association Declaration of Helsinki [17].

### Data collection

A modified version of the Picker Patient Experience Questionnaire (PPE-15) was used to collect data on patients’ perceptions of care. The original PPE-15 is a validated instrument for assessing patient experience in healthcare, and modifications were made to adapt the questionnaire to the EUS context [18, 19]. The questions could be answered on a 5-point Likert Scale or with yes or no in some questions. Patient pre-procedure anxiety, procedural pain, and procedural discomfort were measured using a horizontal visual analogue scale (VAS), labelled only with a descriptor at each end (no pre-procedural anxiety, procedural pain and procedural discomfort to worst possible pre-procedural anxiety, procedural discomfort and procedural pain) (Supplementary File 1). The questionnaire was initially piloted in 15 patients to assess comprehension. The questionnaire included sections on:

- Sociodemographic data
- Indication for EUS
- Previous endoscopy, the experience of any previous endoscopy
- Presence, cause, and level of pre-procedural anxiety
- Quantity and clarity of the written and spoken information delivered to the patient before and after the procedure
- Emotional comfort
- Attitude to patient participation in care
- Maintained dignity and respect during the visit to the endoscopy unit
- Procedural pain and discomfort
- Presence and cause of post-EUS pain and discomfort

The questionnaire was distributed to patients after recovery post-EUS in either paper or digital format, depending on the patient’s ability to use digital devices and required approximately 5–10 min to complete. Staff observed patients’ ability to understand and complete the pre-discharge questionnaire with respect to fatigue and the impact of sedatives after EUS. Patients who were unable or under the influence of sedative drugs were offered a prepaid envelope, including the questionnaire, to return after discharge. Medical indication for EUS and procedure-related data, such as dose of sedatives, procedural time, type of EUS instrument, the performance of fine needle aspiration/biopsy (FNA/FNB), endoscopy findings, and adverse events related to the EUS procedure, were retrieved from the medical reports.

### EUS procedure

The EUS procedure was performed by experienced endosonographers with two nurses present on-site. A therapeutic (Pentax EG-38) or a slim (Pentax EG-34) echoendoscope was used in all procedures. Intravenous opiate analgesics (alfentanil 0.5 mg/ml and/or pethidine 10 mg/ml) and benzodiazepine (midazolam 1 mg/ml) were administered to patients at the discretion of the endosonographer. At the very start of the procedure, and based on patient anxiety, age, and body mass, the patients were given an initial dose of 0.25–0.75 mg of alfentanil, 1–4 mg of midazolam and in some cases 25–50 mg of pethidine. If needed, repeated doses of alfentanil, pethidine, and midazolam were administered during the procedure.

### Definitions and statistical analysis

Pre-procedural anxiety was defined as the anxiety experienced by patients before but not during the very EUS procedure. Procedural pain and discomfort were defined as pain and discomfort experienced by the patients at any time during the EUS procedure but not pain or discomfort experienced before or after EUS. Patient-reported pre-procedural anxiety and procedural pain and discomfort on the VAS scale (range 0–100) were categorised into quartiles and analysed in relation to patient- and procedure-related variables. A binary logistic regression analysis was performed to examine factors associated with pre-procedural anxiety > VAS30. Multicollinearity diagnostics indicated no significant correlations between the independent variables. Logistic regression analyses were not performed for pain or discomfort due to the limited number of cases reporting VAS > 30. Categorical variables are presented as numbers with percentages. Continuous variables are presented as median with interquartile range (IQR). The Kruskal–Wallis test was performed to compare continuous variables, followed by pairwise comparisons to examine differences between quartiles. The Chi-Square Test for independence was performed to test for variances in categorical data. The independent samples t-test was used to test for differences in continuous data. A *p*-value < 0.05 was considered statistically significant. SPSS version 20 (IBM SPSS Incorporation, Chicago, IL, USA) was used for statistical analyses.

## Results

**Fig. 1:**
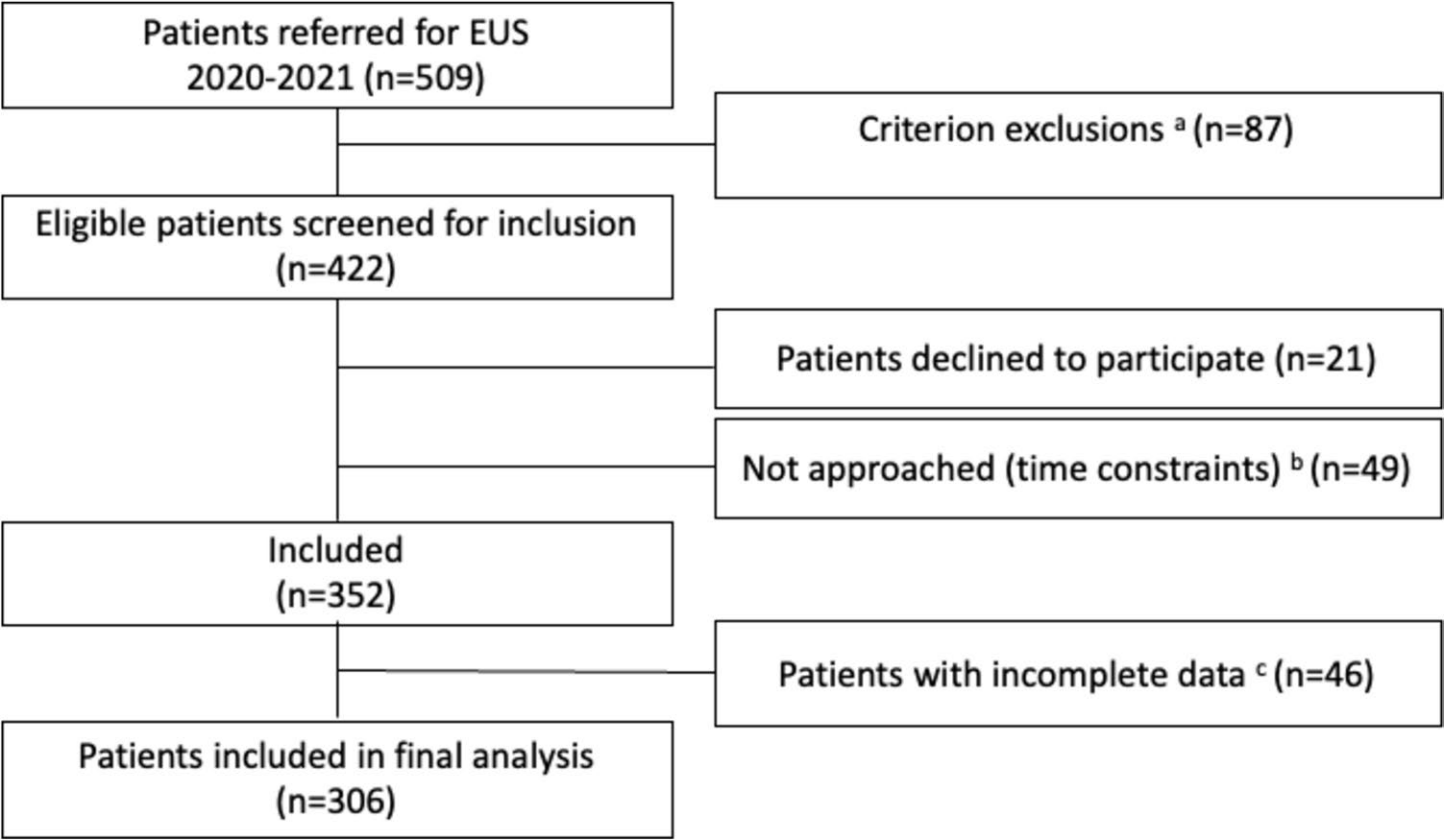
^a^Inpatient EUS (*n* = 47), EUS in general anaesthesia (*n* = 19), patients with poor knowledge in Swedish (*n* = 15), patients with cognitive impairment (*n* = 4), patients previously included in the study (*n* = 2). ^b^Time constraint due to high workload at the endoscopy unit. ^c^Incomplete questionnaire (*n* = 38), missing consent (*n* = 6), incomplete EUS-examination (*n* = 2)

During the study period, 509 examinations were scheduled for EUS. Of the 422 patients who met the inclusion criteria, 306/422 (73%) [median age 69 years; women163/306 (53%)] were included in the final analysis. The remaining 116 patients were eligible but not included due to declining participation (*n* = 21), not being approached because of time constraints, primarily high workload at the endoscopy unit (*n* = 49), or incomplete data (*n* = 46) (Fig. 1). A dropout analysis showed no significant differences in age or sex between the included patients (*n* = 306) and those eligible but not included (*n* = 116). However, a significantly higher proportion of patients in the non-included group had confirmed or suspected malignancy [non-included group: 27/116 (23%), versus included group 47/306 (15%), *p* = 0.035 (Supplementary File 2).

Nearly all patients received conscious sedation with Midazolam 1 mg/ml 302/306 (99%) and Alfentanil 0.1 mg/ml 305/306 (100%). Additionally, 105/306 (34%) received a supplementary Pethidine 50 mg/ml. Previous experience with any form of endoscopy was reported by 224/306 (73%), and 63/306 (21%) had undergone EUS before this study (Table 1). Difficult intubation was reported in 8/306 (3%) cases. In 22/306 (7%) cases, the endosonographer reported that sedation was insufficient to the extent that patient distress negatively impacted the examination. Almost a third of all patients, 91/306 (30%), were examined within the context of a clinical monitoring program, of which 52/306 (17%) were in surveillance for pancreatic cyst lesions.

**Table 1:** Study characteristics

Variable	*n* (%)
Patient characteristics	Median (IQR)
Sex	
Male	143 (47)
Female	163 (53)
Age, years	
Median (IQR)	69 (58–75)
Country of birth	
Nordic countries^a^	271 (89)
Non-Nordic countries	35 (11)
Level of education	
Compulsory school	69 (22)
High school	116 (38)
College/University	121 (40)
Previous exposure to any endoscopy	
Yes	224 (73)
Previous exposure to EUS	
Yes	63 (21)
Previous negative experience of any endoscopy	
Yes	63/224 (28)
Indication for EUS reported by patients^b^	
Surveillance for pancreatic cyst	174 (57)
Suspected malignancy	54 (18)
Multiple indications for EUS	27 (9)
Subepithelial lesion	24 (8)
Unknown by the patient	13 (4)
Abdominal pain	7 (2)
Evaluation of medical treatment	7 (2)
EUS characteristics	
Type of echoendoscope	
Therapeutic	178 (58)
Slim	125 (41)
N/A^c^	3 (1)
Organ of interest	
Pancreas	228 (75)
Subepithelial lesion	54 (18)
Lymph node	11 (4)
Lesion of unclear origin	10 (3)
In the clinical surveillance program^d^	91 (30)
FNA/FNB puncture^e^	
Yes	248 (81)
No	58 (19)
Procedure time, minutes	
All, median (IQR)	27 (22–34)
FNA/FNB, median (IQR)	29 (23–36)
Not FNA/FNB, median (IQR)	19 (12–22)
Alfentanil 0.1 mg/ml	
Yes	305 (100)
Median (IQR)	1.0 (0.75–1.25)
Midazolam 1 mg/ml	
Yes	302 (98)
Median (IQR)	3 (2–4)
Pethidine 50 mg/ml	
Yes	105 (34)
Median (IQR)	50 (25–75)
Complete EUS performed as indented, *n* (%)	301 (98)
Adverse events^f^	16 (5)

Categorical variables are presented as numbers and percentages. Continuous variables are presented as median and interquartile range (IQR)

^a^Nordic country: Sweden, Norway, Denmark, Finland, Iceland

^b^Indication reported by the patients

^c^N/A = non-annotated

^d^surveillance for intraductal papillary mucinous neoplasms (IPMN), pancreatic neuroendocrine tumor (pNET), enterochromaffin-like tumors (ECL)

^e^FNA, Fine needle aspiration; FNB, fine needle biopsy

^f^defines by extended observation time or in-hospital care (pancreatitis *n* = 4, local bleeding *n* = 9, atypical pain *n* = 3

**Fig. 2:**
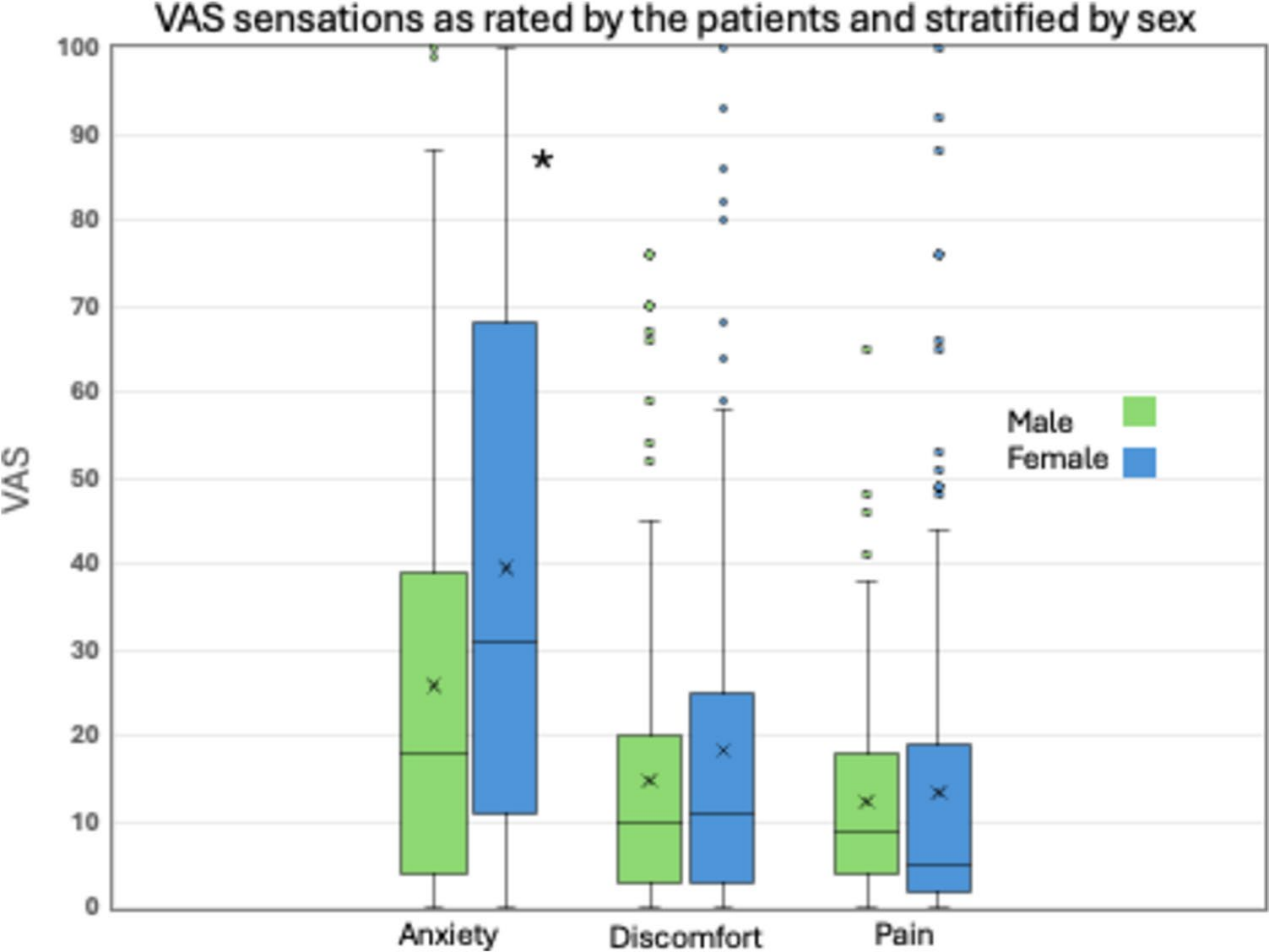
Box plots show median (line), mean (X), and interquartile range (IQR). Differences between sexes were tested using the Mann–Whitney *U* test (median, IQR, *p*-values are shown above). *Indicate statical significance. Anxiety: male 18 (4–39), female 31 (11–68) *p* < .001*. Discomfort: male 10 (3–20), female 11 (3–25) *p* = 0.347. Pain: male 9 (4–18), female 5 (2–19) *p* = 0.069

Pre-procedure anxiety, procedural pain, and procedural discomfort had median VAS scores of 24 (IQR 7–55), 7 (IQR 3–18) and 10 (IQR 3–22), respectively (Fig. 2). Additional median VAS scores for sex, country of birth, level of education, indication for EUS, organ of interest, type of EUS instrument, and FNA/FNB are presented in Supplementary File 3.

### Predictive factors for pre-procedure anxiety

Multivariable analyses showed that sex and negative experience were significantly associated with higher odds of reporting anxiety > VAS30. Females had higher odds of anxiety > VAS30 compared with males (*p* = 0.003). Patients reporting a negative experience from previous edoscopies had more than three times higher odds of anxiety > VAS30 compared to those without a negative experience (*p* < 0.001). Multicollinearity diagnostics indicated no significant correlations between the independent variables. Age and indication for examination (suspected malignancy vs surveillance) were not significantly associated with anxiety levels (*p* = 0.122 and *p* = 0.431, respectively) (Table 2).

**Table 2:** Factors associated with preoperative anxiety, Visual Analogue Scale (VAS > 30): crude and adjusted odds ratios (ORs) with 95% confidence intervals (CI)

Predictors	VAS 0–29.9, *n* (%)		VAS 30–100, *n* (%)		Univariable analysis				Multivariable analysis		
					OR (95% CI)		*p*-value		Adjusted OR (95% CI)		*p*-value
Sex											
Male	96 (67)		47 (33)		reference				2.44 (1.35–4.39)		0.003*
Female	76 (47)		87 (53)		2.34 (1.47–3.73)		< 0.001*				
Age											
Median (IQR)	172 (56) 70 (60–76)		134 (44) 67 (57–73)		0.98 (0.96–1.01)		0.060		0.98 (0.96–1.01)		0.122
Previous negative experience to endoscopy											
No		104 (65)		57 (35)		reference					
Yes		21 (33)		42 (67)		3.65 (1.97–6.75)		< 0.001*		3.58 (1.87–6.87)	< 0.001*
Indication of EUS^a^											
Malignancy^b^	56 (53)		49 (47)		reference						
Surveillance^c^	107 (57)		79 (43)		1.19 (0.732–1.917)		0.489		0.78 (0.43–1.44)		0.431

Categorical variables are presented as numbers and percentages. Continuous variables are presented in medians and interquartile range (IQR)

^a^Indication as reported by the patients

^b^Malignancy, subepithelial lesion, gastrointestinal pain

^c^Surveillance of pancreatic cyst lesion -medical treatment -surgical treatment

*Indicates statistical significance

### The sedation doses across anxiety quartiles

The median dose of midazolam 1 mg/ml differed significantly across quartiles of anxiety (*p* = 0.008). Pairwise comparisons showed that patients in Q3 (*p* = 0.004) and Q4 (*p* = 0.003) received significantly higher doses than those in Q1. The median dose of alfentanil 0.1 mg/ml also differed significantly across quartiles (*p* < 0.001). Pairwise comparisons demonstrated higher doses in Q3 compared with Q1 (*p* = 0.001) and in Q4 compared with Q2 (*p* = 0.035). Overall, an increasing level of pre-procedural anxiety across quartiles was associated with increasing doses of midazolam and alfentanil (Table 3).

**Table 3:** Patients’ estimation of VAS concerning preprocedural ANXIETY, reported in quartiles

Variable	*n* (%) median (IQR)	*n* (%) median (IQR)	*n* (%) median (IQR)	*n* (%) median (IQR)	*p-*value chi-square	*p-*value Kruskal–Wallis
	Quartile 1 *n* = 75	Quartile 2 *n* = 75	Quartile 3 *n* = 79	Quartile 4 *n* = 77		
VAS^a^	0–7	7.01–24	24.01–55	> 55.01		
Sex						
Male	46 (61)	37 (49)	37 (47)	23 (30)		
Female	29 (39)	38 (51)	42 (53)	54 (70)	0.001*	
Age, median (IQR)	71 (60–77)	70 (60–75)	66 (56–75)	68 (60–72)		0.103
Indication^b^						
Suspected malignancy^c^	23 (32)	22 (31)	32 (43)	28 (38)		
Surveillance of previous findings^d^	48 (68)	49 (69)	43 (57)	46 (62)	0.436	
Previous exposure to any endoscopy						
Yes	58 (77)	49 (65)	64 (81)	53 (69)		
No	17 (23)	26 (35)	15 (19)	24 (31)	0.101	
Previous negative experience with any endoscopy						
Yes	11 (19)	5 (10)	22 (34)	25 (47)		
No	47 (81)	44 (90)	42 (66)	28 (53)	< 0.001*	
Positive attitude toward patient participation						
Yes	16 (31)	15 (27)	17 (34)	15 (31)		
No	35 (68)	40 (73)	33 (66)	34 (69)	0.903	
Alfentanil 0.1 mg/ml, median (IQR)	0.75 (0.5–1.0)	0.75 (0.5–1.0)	1.0 (0.75–1.25)	1.0 (0.75–1.25)		< 0.001*
Midazolam 1 mg/ml, median (IQR)	2 (2–3)	2 (2–4)	3 (2–4)	3 (2–4)		0.008*
Pethidine 50 mg/ml						
Yes	23 (31)	25 (33)	24 (30)	33 (43)		
No	52 (69)	50 (67)	42 (70)	44 (57)	0.319	

Categorical variables are presented as numbers and percentages. Continuous variables are presented in medians and interquartile range (IQR)

^a^Visual analogue scale

^b^Indication as reported by the patients

^c^Malignancy, subepithelial lesion, gastrointestinal pain

^d^Surveillance of pancreatic cyst lesion -medical treatment -surgical treatment

*Indicates statistical significance

### Predictive factors for procedural pain

Increasing levels of procedural pain across quartiles were associated with a higher prevalence of female gender (*p* < 0.005) and negative experiences of previous endoscopy (*p* = 0.004). Median age differed significantly across quartiles (*p* = 0.012), and pairwise comparisons showed that patients in Q3 were significantly younger than those in Q1 (*p* = 0.01) (Table 4).

**Table 4:** Patients’ estimations of VAS concerning procedural PAIN, reported in quartiles

Variable	*n* (%) median (IQR)	*n* (%) median (IQR)	*n* (%) median (IQR)	*n* (%) median (IQR)	*p-*value chi-square	*p-*value Kruskal–Wallis
	Quartile 1 (*n* = 99)	Quartile 2 (*n* = 59)	Quartile 3 (*n* = 72)	Quartile 4 (*n* = 76)		
VAS^a^	0–3.0	3.01–7.0	7.01–18.25	> 18.26		
Sex						
Male	33 (33)	33 (56)	42 (58)	35 (46)		
Female	66 (66)	26 (44)	30 (42)	41 (54)	< 0.005*	
Age, median (IQR)	71 (63–77)	68 (56–75)	65 (52–72)	70 (56–75)		0.012*
Indication^b^						
Suspected malignancy^c^	37 (40)	15 (26)	26 (38)	27 (37)		
Surveillance of previous findings^d^	55 (60)	42 (74)	43 (72)	46 (73)	0.370	
Previous exposure to any endoscopy						
Yes	66 (67)	41 (70)	57 (79)	60 (79)		
No	33 (33)	18 (30)	15 (21)	16 (21)	0.161	
Previous negative experience with any endoscopy						
Yes	11 (17)	10 (76)	42 (74)	27 (45)		
No	55 (83)	31 (24)	15 (26)	33 (55)	0.004*	
Positive attitude toward patient participation						
Yes	20 (29)	14 (31)	11 (26)	18 (37)		
No	48 (71)	31 (69)	32 (74)	31 (63)	0.700	
Procedure time, minutes, median (IQR)	25 (20–33)	26 (21–31)	28 (19–31)	29 (21–39)		0.349
FNA/FNB^e^						
Yes	82 (83)	42 (71)	62 (86)	62 (82)		
No	17 (17)	17 (29)	10 (14)	14 (18)	0.161	
Type of echoendoscope						
Therapeutic	48 (49)	39 (67)	42 (59)	49 (65)		
Slim	50 (51)	19 (33)	29 (41)	27 (35)	0.085	
Midazolam 1 mg/ml, median (IQR)	3 (2–4)	3 (2–4)	3 (2–3)	3 (2–4)		0.648
Alfentanil 0.1 mg/ml, median (IQR)	0.75 (0.75–1.0)	0.75 (0.75–1.25)	1.0 (0.5–1.25)	1.0 (0.75–1.25)		0.643
Pethidine 50 mg/ml						
Yes	40 (40)	26 (44)	21 (29)	18 (24)		
No	59 (60)	33 (56)	51 (71)	58 (76)	0.032*	

Categorical variables are presented as numbers and percentages. Continuous variables are presented in medians and interquartile range (IQR)

^a^Visual Analogue Scale

^b^Indication as reported by the patients

^c^Malignancy, subepithelial lesion, gastrointestinal pain

^d^Surveillance of pancreatic cyst lesion -medical treatment -surgical treatment

^e^Fine needle puncture/fine needle biopsy

*Indicates statistical significance

### Sedation doses across pain quartiles

Administration of an additional dose of pethidine (10 mg/mL) was more common among patients reporting lower levels of procedural pain, reflected by a decreasing proportion of patients receiving additional pethidine across increasing pain quartiles (*p* = 0.032) (Table 4).

### Predictive factors for procedural discomfort

The prevalence of previous negative endoscopy experiences increased across higher quartiles of procedural discomfort (*p* = 0.05). Median age also differed significantly between quartiles (*p* = 0.049), with patients in Q3 (*p* = 0.048) and Q4 (*p* = 0.015) being significantly younger than those in Q1, indicating decreasing age with increasing discomfort quartiles (Table 5).

**Table 5:** Patients’ estimations of VAS concerning procedural DISCOMFORT, reported in quartiles

Variable	*n* (%) median (IQR)	*n* (%) median (IQR)	*n* (%) median (IQR)	*n* (%) median (IQR)	*p-*value chi-square	*p-*value Kruskal–Wallis
	Quartile 1 (*n* = 90)	Quartile 2 (*n* = 64)	Quartile 3 (*n* = 76)	Quartile 4 (*n* = 76)		
VAS^a^	0–3.0	3.01–10.0	10.01–22.25	> 22.26		
Sex						
Male	43 (48)	31 (48)	37 (49)	32 (42)		
Female	47 (52)	33 (52)	39 (51)	44 (58)	.829	
Age, median (IQR)	71 (62–75)	70 (63–75)	67 (56–74)	66 (50–74)		.049*
Indication for EUS^b^						
Suspected malignancy^c^	27 (32)	19 (32)	29 (40)	30 (40)		
Surveillance of previous findings^d^	57 (68)	41 (68)	44 (60)	44 (60)	.546	
Previous exposure to any endoscopy						
Yes	60 (67)	46 (72)	54 (71)	64 (84)		
No	30 (33)	18 (28)	22 (29)	12 (16)	.075	
Previous negative experience with any endoscopy						
Yes	10 (17)	11 (24)	18 (33)	24 (37)		
No	50 (83)	35 (76)	36 (67)	40 (63)	.05*	
Positive attitude toward patient participation						
Yes	22 (34)	10 (20)	12 (26)	19 (42)		
No	43 (66)	39 (80)	34 (74)	26 (58)	.111	
Procedure time, minutes, median (IQR)	25 (20–33)	28 (21–34)	28 (20–35)	28 (20–37)		.883
FNA/FNB^e^						
Yes	71 (79)	53 (17)	61 (80)	63 (83)		
No	19 (21)	11 (82)	15 (20)	13 (17)	.896	
Type of echoendoscope						
Therapeutic	47 (52)	43 (67)	44 (57)	47 (62)		
Slim	43 (48)	21 (33)	32 (43)	29 (38)	.557	
Midazolam 1 mg/ml, median (IQR)	3 (2–4)	2.5 (2–4)	3 (2–4)	3 (2–4)		.771
Alfentanil 0.1 mg/ml, median (IQR)	0.75 (0.75–1.25)	1.0 (0.75–1.25)	1.0 (0.5–1.25)	1.0 (0.75–1.25)		.273
Pethidine 50 mg/ml						
Yes	35 (61)	23 (64)	27 (65)	20 (74)		
No	55 (39)	41 (36)	49 (35)	56 (26)	.374	

Categorical variables are presented as numbers and percentages. Continuous variables are presented in medians and interquartile range (IQR)

^a^Visual analogue scale

^b^Indication as reported by the patients

^c^Malignancy, subepithelial lesion, gastrointestinal pain

^d^Surveillance of pancreatic cyst lesion -medical treatment -surgical treatment

^e^Fine needle puncture/fine needle biopsy

*Indicates statistical significance

### Patient-reported experience measures (PREM)

Completion rates were high (> 95%) for individual questions. A total of 63/306 (21%) patients expressed a desire to be more actively involved in their care, while 101/306 (33%) expressed a neutral position to the question *“I would have liked to be more involved in decisions regarding my care and treatment*” (Fig. 2). The outcome of the EUS-examination was the most frequently reported cause of concern to patients, reported in 150/305 (49%) cases. Other causes of concern mentioned by patients included *‘the gag reflex’*, ‘*the urge to vomit’*, and ‘*the insertion of the instrument’*. Post-procedural discomfort/pain was reported by 183/306 (60%) patients, of which 111/306 (36%) experienced pharyngeal discomfort/pain, and 31/306 (10%) abdominal discomfort/pain. Post-procedural pharyngeal discomfort/pain was reported by 71/178 (40%) of patients examined with a therapeutic EUS instrument and by 39/125 (31%) (*p* = 0.12) of those examined with a slim instrument. A total of 302/306 patients (99%) reported that care before, during, and after the EUS procedure was provided with dignity and respect.

## Discussion

In this large cross-sectional study of patients undergoing EUS with conscious sedation, a previous negative endoscopy experience was the strongest predictor of higher pre-procedural anxiety, followed by female gender. These factors were also more frequent among patients reporting higher levels of procedural pain and discomfort, increasing across quartiles. Younger age was another significant factor, being more prevalent in the higher quartiles of discomfort. Previous studies examining patient experiences with endoscopy (gastroscopy, colonoscopy) are consistent with these findings and may reflect established psychological mechanisms [5, 20–22]. However, the procedural tolerance of EUS in the present study was high, with a median VAS score of 10 for discomfort and 7 for pain.

High levels of pre-procedural anxiety were associated with an increased need for intravenous sedation (imidazolam 1 mg/ml and alfentanil 0.1 mg/ml) suggesting that increased anxiety may complicate the sedation management [5, 7]. Lauriola et al. showed that anxiety, sensitivity, and worry are associated with following clinical outcomes in the pain domain, which is consistent with our observation that women who report higher levels of anxiety also report higher levels of pain [5]. While dose escalation may improve patient comfort, it also carries risks, particularly in the predominantly older EUS population, where pharmacodynamic sensitivity varies and must be taken into account [8]. Furthermore, higher levels of discomfort were reported among younger age, which may indicate a need for a more individualised sedation strategy in this group [23, 24]. Propofol has been suggested as an effective alternative to reduce discomfort while maintaining procedural quality [25, 26]. Still, its wider use in Europe remains limited due to resource constraints and shortages of anaesthetic personnel [26].

Fear of malignant disease emerged as the most prevalent concern in our study, contrasting with studies on gastroscopy and colonoscopy, where fear of procedural pain has been identified as the primary source of worry [21, 27, 28]. This difference likely reflects the typical indication for EUS, to diagnose a probable malignancy or for surveillance of premalignant lesions. Also, a previous negative endoscopic experience with endoscopy was associated with higher pain, consistent with earlier studies [7, 29], which highlight the importance of optimising care during EUS since many patients undergo EUS in the context of diagnosing serious illness or as a part of long-term surveillance. Consequently, negative endoscopy experience may lead not only to increased discomfort and pain during EUS but may also negatively affect adherence and complicate follow-up procedures, underscoring the importance of addressing these findings [7, 16].

The integration of EUS into routine clinical practice is supported by the requirement for minimally invasive techniques for shorter recovery time and lower procedural risk, especially for the elderly and vulnerable group of patients [30]. As EUS continues to expand beyond diagnostics with therapeutic interventions such as EUS-guided drainage and tumour radiofrequency ablation, the clinical role in endoscopy is increasing, further underscoring the importance of understanding procedure-related patient experience [31]. No therapeutic interventions were made on our study population. Still, diagnostic sampling via EUS-FNA/FNB was common and did not show any significant difference in discomfort or pain between sampled and non-sampled patients. Although procedure tolerance with EUS was high, post-procedural pharyngeal or abdominal discomfort/pain was frequently reported, suggesting an effect of the EUS instrument’s large diameter and a rigid tip.

While highly standardised procedures, such as EUS, are effective and routinely performed, they may limit flexibility to adapt care to individual patient needs [32]. Person-centred care, tailoring communication and involvement to patient preferences, has been associated with higher patient satisfaction, better adherence, and fewer adverse events [1, 33, 34]. Accordingly, the ESGE Quality Improvement Committee highlights the importance of measuring and evaluating procedural quality and patient experience [34]. Furthermore, the increasing interest in using PREMs reflects a shift in care towards person-centred endoscopic practice [10, 35, 36]. Few patients expressed a desire for greater involvement in their care. This may reflect the technically complex environment of EUS and perceived power imbalance, a preference for a more guided role, or that patients already experience involvement as sufficient, as most described care as dignified and respectful [37]. Participation is also shaped by context; in the endoscopy setting, Dubois et al. highlight that clear information and explanations throughout the care pathway are central aspects for patient participation [37]. Structured tools such as person-centred checklists may support appropriate information delivery and offer opportunities for patient involvement during EUS [38]. Regular use of PREM in endoscopic care may therefore help identify areas where communication, information, or support can be improved, thereby contributing to ongoing quality improvement.

Despite the growing interest in EUS in clinical practice, there is a notable lack of studies assessing performance outcomes and PREMs specific to EUS [35]. EUS is known as one of the most technically demanding endoscopic procedures, requiring substantial training and expertise to become a professional endosonographer [39]. Previous studies within the context combine different endoscopic procedures without stratifying the setting or clearly defining sedation protocols, which may limit the interpretation of EUS-specific outcomes [11–13]. The integration of self-reported and medical data to enable a comprehensive understanding of the patient’s experience and procedural details is a strength in the present study. The procedure-specific data from a well-defined outpatient study group under standardised conscious sedation is thereby contributing important knowledge to the field.

Methodological considerations should be acknowledged. The VAS scale was used to assess anxiety, pain, and discomfort for capturing subjective experiences across a broad range [40, 41]. However, pain and discomfort are distinct but interrelated sensations, including physical and psychological dimensions that were not separately analysed in the study, highlighting multidimensional instruments in future studies [21, 42]. Furthermore, the overall positive PREM responses suggest a potential ceiling effect, which should be considered when interpreting the results [43, 44]. The questionnaires were distributed after the procedure, and to account for possible post-procedural fatigue, some patients were provided with prepaid return envelopes to complete the questionnaire later; however, many of these were not returned. Nevertheless, the overall response rate remained high compared with similar studies, which strengthens the reliability of the findings [12]. Sedation with midazolam and alfentanil may have induced short-term amnesia and influenced PREM responses; however, doses were generally low, and patients remained responsive, consistent with standard conscious sedation practice. The relatively high number of patients is a strength of our study. Nevertheless, the number of patients reporting significant (VAS > 30) pain or discomfort was limited, preventing the use of logistic regression for this outcome. Finally, the single-centre study design may limit generalisability, and the lack of a validated EUS-specific instrument for assessing patient experience could have led to procedure-related aspects such as intubation and procedural management being overlooked [35].

We suggest that our findings have direct implications for clinical practice. Early identification of patients at increased risk of procedure-related anxiety and discomfort may enable proactive and individualised management aimed at optimising comfort and quality during EUS. Such management could include the use of screening tools to support tailored communication, information, and reassurance before and under the procedure [36, 45].

## Conclusion

Our results show that routine EUS is generally well tolerated with minimal pain and discomfort during conscious sedation. However, negative experiences from previous endoscopic procedures, female gender and younger age were associated with higher pre-procedure anxiety and increased procedural discomfort and pain. Our study provides important and clinically relevant information that can be used to increase awareness of patients at increased risk of anxiety and discomfort and design person-centred care with tailored information and reassurance adapted to individual needs and preferences.

## Supplementary Information

Below is the link to the electronic supplementary material.

Supplementary file1 (DOCX 31 KB)

Supplementary file2 (DOCX 39 KB)

Supplementary file3 (DOCX 19 KB)

## Data Availability

Data described in the manuscript, code book, and analytic code will be made available upon request.
