# Supplementary material for: Factors influencing the patient experience of gastrointestinal endoscopic ultrasound: a Swedish cross-sectional study
Source: Surg Endosc. 2026 May 15;40(7):6030–41. doi: 10.1007/s00464-026-12777-7 (PMC13368844; doi:10.1007/s00464-026-12777-7)
Supplement: Supplementary file 1 — Supplementary file1 (DOCX 31 KB) [file 464_2026_12777_MOESM1_ESM.docx]

**Supplementary file 1.** Patient-reported experience measure questionnaire (PREM)

1. State your study ID

2. Enter your gender

3. Enter your age in years

4. Enter your country of birth:

Sweden

Another country in the Nordic region

Another country in Europe

Another country outside Europe

5. Enter your highest level of education

Compulsory school

High School

College/University

6. Have you had any endoscopic examination before?

Yes

No

7. What endoscopic examination have you had before?

Gastroscopy

Colonoscopy

ERCP- biliary tract examination

Endoscopic ultrasound examination

8. Do you have any unpleasant experiences with previous endoscopic examinations?

Yes

No

9. For what reason should you undergo examination today?

*You can choose 1 option. Choose the most suitable reason.*

Investigation of suspected serious illness

Control of pancreatic cysts

Control of medical treatment

Control of a lump in the gastrointestinal tract

Stomachache

I don't know

Other, reply in the text field

10. State your perceived anxiety before the examination on the scale below

***Estimate your anxiety by adjusting the arrow on the scale***

No anxiety Worst possible anxiety

I I

11. Before the examination, I was worried about:

*Several response options are possible. You can choose from 1 to 4 options.*

I had no worries

That it was going to be painful

That it should be difficult to breathe

That I was unable to control the situation

That I was in an environment where I felt unsafe

The outcome of the survey

Other, reply in the text field

12. The written information sent to me before the examination was easy to understand

Totally agree

Partially agree

Neither agree nor disagree

Partially disagree

Partially disagree

I didn't achieve any written information

13. The written information sent to me answered my questions

Totally agree

Partially agree

Neither agree nor disagree

Partially disagree

Totally disagree

I didn't achieve any written information

14. The verbal information given to me before the examination was easy to understand

Totally agree

Partially agree

Neither agree nor disagree

Partially disagree

Totally disagree

15. The oral information given to me before the examination answered my questions

Totally agree

Partially agree

Neither agree nor disagree

Partially disagree

Totally disagree

16. Please indicate your perceived pain during the examination on the pain scale below

***Estimate your pain by adjusting the arrow on the scale***

No pain Worst possible pain

I I

17. When I was in pain during the examination, the staff did everything they could to relieve my pain

Totally agree

Partially agree

Neither agree nor disagree

Partially disagree

Totally disagree

I didn’t have any pain

17. Please indicate your perceived discomfort during the examination

***Estimate your discomfort by adjusting the arrow on the scale***

No discomfort Worst possible discomfort

I I

19. After the examination, I experienced pain and/or discomfort

Totally agree

Partially agree

Neither agree nor disagree

Partially disagree

Totally disagree

20. After the examination, I felt pain/discomfort in

In my throat

In my stomach

In the back

Other: enter the free text section

21. Did healthcare professionals talk to each other in your presence as if you weren't there?

Totally agree

Partially agree

Neither agree nor disagree

Partially disagree

Totally disagree

22. If you asked a nurse important question, did you get answers that you could understand?

Totally agree

Partially agree

Neither agree nor disagree

Partially disagree

Totally disagree

I didn’t have any question

23. If you asked a doctor important question, did you get answers that you could understand?

Totally agree

Partially agree

Neither agree nor disagree

Partially disagree

Totally disagree

I didn’t have any question

24. At the hospital, it happened that a doctor or nurse says one thing while another said a completely different thing on the same matter

Totally agree

Partially agree

Neither agree nor disagree

Partially disagree

Totally disagree

25. When I was worried about my condition or your treatment, I was supported by a nurse

Totally agree

Partially agree

Neither agree nor disagree

Partially disagree

Totally disagree

I had no worries

26. When I was worried about my condition or your treatment, I was supported by a doctor

Totally agree

Partially agree

Neither agree nor disagree

Partially disagree

Totally disagree

I had no worries

27. The verbal information given about the results of the survey was easy to understand

Totally agree

Partially agree

Neither agree nor disagree

Partially disagree

Totally disagree

28. The written information provided on the results of the examination was easy to understand

Totally agree

Partially agree

Neither agree nor disagree

Partially disagree

Totally disagree

29. The information regarding the follow-up after the examination was easy to understand

Totally agree

Partially agree

Neither agree nor disagree

Partially disagree

Totally disagree

30. I would have liked to have been more involved in decisions regarding my care and treatment

Totally agree

Partially agree

Neither agree nor disagree

Partially disagree

Totally disagree

31. During the visit, I was treated with dignity and respect by the medical staff

Totally agree

Partially agree

Neither agree nor disagree

Partially disagree

Totally disagree
