# Supplementary material for: Factors influencing the patient experience of gastrointestinal endoscopic ultrasound: a Swedish cross-sectional study
Source: Surg Endosc. 2026 May 15;40(7):6030–41. doi: 10.1007/s00464-026-12777-7 (PMC13368844; doi:10.1007/s00464-026-12777-7)

Supplementary file 2

**Figure legend:**

^a^ Time constraint, due to high workload at the endoscopy unit.

^b^ Incomplete questionnaire (n=38), missing consent (n=6), incomplete EUS-examination (n=2).


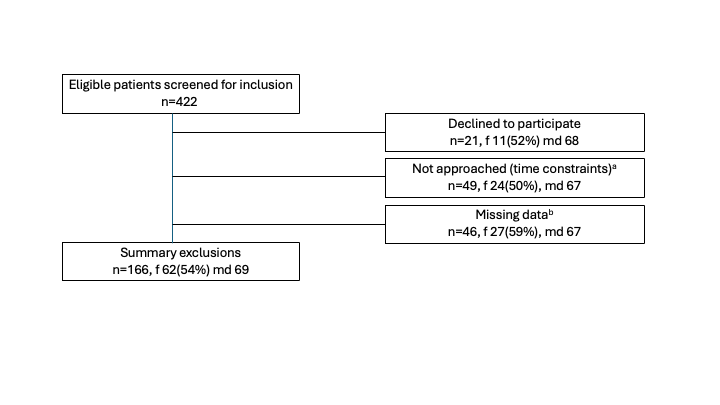

Supplement: Supplementary file 2 — Supplementary file2 (DOCX 39 KB) [file 464_2026_12777_MOESM2_ESM.docx]
