# Supplementary material for: Factors influencing the patient experience of gastrointestinal endoscopic ultrasound: a Swedish cross-sectional study
Source: Surg Endosc. 2026 May 15;40(7):6030–41. doi: 10.1007/s00464-026-12777-7 (PMC13368844; doi:10.1007/s00464-026-12777-7)
Supplement: Supplementary file 3 — Supplementary file3 (DOCX 19 KB) [file 464_2026_12777_MOESM3_ESM.docx]

| **Supplementary file 3**. VAS sensations as estimated by the patients in groups of variables in median and (IQR) | | | | |
| --- | --- | --- | --- | --- |
| Variable |  | **Anxiety** median (IQR) | **Pain**  median (IQR) | **Discomfort**  median (IQR) |
| **Sex** | Male | 18 (4 - 39) | 9 (4 - 18) | 10 (3 - 20) |
|  | Female | 31 (11 - 68) | 5 (2 - 19) | 11 (3 - 25) |
| **Country of birth** | Nordic Country | 24 (7–58) | 7 (3 - 18) | 10 (3–24) |
|  | Non- Nordic Country | 19 (4–45) | 18 (3 - 19 | 13 (4–19) |
| **Level of Education** | Compulsory school | 20 (5–56) | 4 (2–19) | 8 (3–19) |
|  | High school | 22 (6–53) | 8 (3–20) | 9 (3–23) |
|  | College/University | 28 (8–60) | 7 (3-18) | 13 (4–27) |
| **Indication** | Suspected malignity  (prior 2v) | 24 (5–57) | 8 (3–22) | 11(3–23) |
|  | Surveillance | 26 (6–51) | 6 (3–16) | 13 (4–22) |
|  | Remaining indications | 24 (7–54) | 7 (3–18) | 10 (3–22) |
| **Organ of interest** | Pancreas | 24 (7-55) | 7 (3-18) | 10 (3-22) |
|  | Lymph nodes | 16 (4-66) | 5 (3-16) | 3 (0-20) |
|  | Subepithelial lesion | 26 (4-55) | 19 (3-18) | 11 (4-27) |
|  | Detached tumors | 21 (7-29) | 4 (2-22) | 7 (3-19) |
| **Type of scope** | Therapeutic | 26 (9–58) | 8 (3–21) | 11(3–25) |
|  | Standard | 19 (4–52) | 5 (3–17) | 10 (3–21) |
| **Punction** | Yes | 22 (6–58) | 8 (3–19) | 11 (3–23) |
|  | No | 29 (11–52) | 6 (3–18) | 10 (3–19) |
